# Determinants and Variations of COVID-19 Vaccine Uptake and Responses Among Minority Ethnic Groups in Amsterdam, the Netherlands

**DOI:** 10.3389/fpubh.2022.761987

**Published:** 2022-02-17

**Authors:** Daniel Antwi-Berko, Amisah Zenabu Bakuri, Kenneth Bentum Otabil, Alexander Kwarteng

**Affiliations:** ^1^Department of Basic and Applied Biology, University of Energy and Natural Resources (UENR), Sunyani, Ghana; ^2^Amsterdam Institute of Social Science Research (AISSR), University of Amsterdam, Amsterdam, Netherlands; ^3^Department of Biochemistry and Biotechnology, Kwame Nkrumah University of Science and Technology, Kumasi, Ghana

**Keywords:** minority ethnic groups, COVID-19 vaccine, vaccine efficacy, vaccine hesitancy, perception

## Abstract

The outbreak of the deadly novel coronavirus disease (COVID-19) has disrupted life worldwide in an unprecedented manner. Over the period, scientific breakthroughs have resulted in the rollout of many vaccination programmes to protect against the disease, reduce the fear and ease public health restrictions for lives to return to some normalcy. The aim of this study was to identify the factors responsible for COVID-19 vaccine acceptance or vaccine hesitancy and to develop a framework to improve vaccine uptake in the Ghanaian-Dutch, Afro and Hindustani Surinamese-Dutch communities in Amsterdam. Using a mixed method approach, this community-based cross-sectional survey recruited 160 respondents consisting of 57 Ghanaian-Dutch, 54 Afro Surinamese-Dutch and 49 Hindustani-Dutch residents in Amsterdam. Our findings showed that the choice of a vaccine as well as the likelihood of self-reported willingness to receive a vaccine is highly dependent on vaccine efficacy and safety. Available evidence of high vaccine effectiveness and safety could encourage about 41.3% of the respondents to accept the vaccine. Additionally, 69.6% of the respondents indicated their willingness to accept the vaccine when vaccine passports are made mandatory by the government. Other major factors that could drive the likelihood of accepting the COVID-19 vaccine include travel requirement for vaccination (28.3%), the safety/probability of only minor side effects (26.1%) and recommendation by family and friends (15.2%). The study therefore provides systematic evidence of factors associated with individual preferences toward COVID-19 vaccination. It demonstrates that the needs of each community are unique and specific interventional efforts are urgently needed to address concerns likely to be associated with vaccine hesitancy.

## Introduction

The novel coronavirus disease 2019 (COVID-19) is a deadly respiratory and systemic disease caused by the Severe Acute Respiratory Syndrome Coronavirus 2 (SARS-CoV2), which has infected over 210 million people and unfortunately killed over 4 million globally as of August 18th 2021 (1). About two thirds of people with COVID-19 are asymptomatic, but other individuals may become severely ill and require hospitalization with respiratory support (2, 3). Human-to-human transmission of COVID-19 occurs through respiratory droplets, contaminated objects, direct physical contact with infected people, and potentially aerosols (4).

At the early onset of the pandemic, the World Health Organization (WHO) strongly recommended countries to implement interventions to curb the rapid spread of COVID-19 through minimizing contact between infected and uninfected persons (4). So far these measures have included lockdowns, closure of educational institutions and public places, banning of large gatherings, self-isolation/quarantine, shielding of the individuals most vulnerable to the infection, use of personal protection equipment (including use of face masks), and stringent personal methods of hand hygiene and physical distancing (5). These measures were necessary at the onset of this novel pandemic to help health systems and policymakers adopt strategies to tackle the virus adequately (5).

The COVID-19 pandemic is arguably the biggest global health crisis of the twenty-first century (6). It continues to impose enormous strain on healthcare systems and plunges nations to a standstill with an unprecedented social and economic impact worldwide (6). Considering that vaccine development is the most effective strategy to prevent and eliminate infectious diseases (7), the global health community began work in earnest with regards to COVID-19 vaccines. Recently, the Pfizer/BioNTech reached a critical milestone in the vaccine development programme at a time when the infection rates continue to rise and stretch hospitals beyond their capacities amidst prolonged lockdowns and economies struggling to reopen (8). With the recent breakthrough development of COVID-19 vaccines by several manufacturers, governments across the globe are in a race against time to acquire and rollout large-scale vaccinations of its population. However, this will require strategies and frameworks that promote peoples' trust in and the acceptance of the vaccine.

Despite the availability of COVID-19 vaccines, generally vaccination has continued to be a subject of many different controversies and vaccination scares. These controversies have affected vaccine acceptance to varying degrees and led to rising incidence of vaccine hesitancy worldwide, particularly among minority groups. Vaccine hesitancy ranked among the 2019 top ten global health threats and is often characterized by a delay or refusal of vaccines despite the availability of vaccination services (9). Though the reasons for vaccine hesitancy are multifaceted, a prominent factor may be the proliferation of conspiracy theories and misinformation arising from several sources particularly anti-vaccination activists. Until this is addressed, it poses an enormous threat to achieving coverage and community immunity. Therefore, there is the need for more research to address this hesitancy and identify potential factors that could build public trust to accept the vaccination programmes.

The COVID-19 pandemic has further illustrated that vaccine research, development, and rollout is crucial for combating emerging infectious disease outbreaks, but has also revealed many unanticipated side effects, ethical, behavioral and inequality questions. We hypothesize that vaccine hesitancy is prevalent among minority ethnic populations and might vary from the Ghanaian-Dutch, Afro and Hindustani Surinamese-Dutch communities in Amsterdam. Available evidence from the most successful countries in terms of COVID-19 vaccines rollout and percentage of population vaccinated against COVID-19 indicates that the uptake of the vaccination programmes is lower among people from minority ethnic groups (10, 11). Scientists have been attempting to understand contemporary vaccine hesitancy leading to refusal, particularly by focusing on the influences around how people make decisions. Sobo (12) has demonstrated how vaccine “refusers” in schools in the United States are not homogenous, showing how hesitancy can lead to selective refusal of vaccines or doses, and hesitancy can be traced along nuanced roots of efficacy, adverse reactions, as well as the broader political and economic culture of vaccine products, processing and procurement.

This present study attempted to identify and understand public perceptions and decision making among the Ghanaian-Dutch, Afro and Hindustani Surinamese-Dutch people resident in Amsterdam regarding COVID-19 vaccine. This is because vaccine hesitancy might partly be fuelled by inadequate knowledge about the prevalence and actual burden of the disease. Unaddressed concerns about the safety of a vaccine could contribute to vaccine hesitancy. It could also be influenced by the lack of confidence of the vaccines and complacency regarding the need for vaccination, and the perception of how conveniently it can be obtained (13). In this study therefore, we plan to investigate the driving factors and causes of vaccine hesitancy with reference to the uptake of COVID-19 vaccines, and develop a framework to mitigate vaccine hesitancy in these communities. This is because mass vaccination programmes and its acceptability are shaped by context (e.g., cultural connotations, history of previous vaccine programmes and availability at community-based health interventions) in addition to individual and vaccine-specific factors (e.g., perceptions often vary by vaccine) (14, 15). We attempt to evaluate the potential vaccination compliance rates among the minority ethnic populations in Amsterdam.

Vaccine hesitancy remains a complex public health issue referring to concerns about the safety, efficacy or need for vaccination. Relatively little is known about vaccine hesitancy among these minority ethnic groups. We aim to assess the sociocultural determinants of vaccine hesitancy regarding COVID-19 vaccines among the three communities in Amsterdam. Our aim is to provide a detailed characterization of vaccine hesitancy and the sociocultural factors to assist policy developers in designing an intervention tailored to improve COVID-19 vaccination among people from minority ethnic groups in Amsterdam. It is against this background that we explore some issues relating to peoples acceptance, refusal or delay regarding the COVID-19 vaccines.

## Materials and Methods

### Study Design

This was a pilot study that applied a mixture of quantitative and qualitative methods to investigate the spectrum/factors responsible for vaccine hesitancy and develop a framework aimed at improving vaccine uptake in the Ghanaian-Dutch, Afro and Hindustani Surinamese-Dutch communities in Amsterdam. The application of these mixed methods allowed for the triangulation of the data to increase its accurateness. This was a community-based cross-sectional survey study and in-depth interviews conducted from January 30th to April 30th 2021.

### Study Area and Population

The study was conducted in the city of Amsterdam, the capital and most populous city of the Netherlands. The metropolitan region of Amsterdam has an estimated population of 2,480,393 and home to many non-Dutch immigrants who either settled for economic reasons or as asylum seekers and undocumented migrants (16). Available statistics show that native Dutch residents constitute about 46.6%, while the remaining trace their routes through immigration (17). Among these minority ethnic groups in Amsterdam are people with Surinamese and Ghanaian backgrounds.

According to current figures as published by the Statistics Netherlands, there are 356,402 people of Surinamese origin making up nearly 2.1% of the Dutch population (18). Out of this Surinamese population, 176,963 belong to the first generation, while 179,439 representing more than half of them were born in the Netherlands. Surinamese with an African background (referred to as Afro Surinamese or “Creole” in the Dutch context) are mainly the descendants of West Africans, and those with a South-Asian background (referred to as “Hindustani” in the Dutch context) have their roots in North India (19). It is also estimated that there are about 12,184 persons who trace their route from Ghana and live within the municipality of Amsterdam (14). Available records showed that about half of the officially registered people with Ghanaian and Surinamese background reside in the Bijlmermeer (popularly known as Bijlmer), a suburb of Zuid Oost (Southeast) municipality (20).

The Zuid Oost municipality is ethnically highly diverse, and it is often referred as Amsterdam city's “black neighborhood” due to the presence of many African migrants (21). Numerous shops sell foods, articles, and fabrics from Ghana and Suriname. It is also an area where several beauticians and hairdressers with diverse background are located. The Ghanaian-Dutch as well as the Surinamese-Dutch residents form a closely-knit community and are predominantly religious (22).

### Ethics Considerations

Sampling for this study was part of a bigger research project “Sexual well-being and relationships among migrants from sub-Sahara Africa in the Netherlands”. This study was approved by Amsterdam Institute for Social Science Research (AISSR) the Ethics Advisory Board of the University of Amsterdam, the Netherlands. Respondents were informed about the purpose, nature and procedures of the survey and the in-depth interviews. No respondent was coerced to participate in this survey and all those who responded did so willingly. Likewise, all respondents who took part in the in-depth interviews willingly consented to be part of this study. There were no personal identifications requested as part of this survey. All names used in this report are pseudonyms.

### Selection and Recruitment of Study Respondents

The target populations for this study were people with a Ghanaian or Surinamese background residing in Amsterdam. It included all persons aged 18 years and above. Respondents were recruited through personal invitations on the streets, from churches, online social media platforms, community parks, and snowballing. Most of the respondents were recruited at hairdressing saloons/shops, marketplaces, and workplaces.

### Study Procedure and Data Collection

#### Quantitative Procedures

##### Sample Size Justification

Sample size for this study was estimated using G^*^ Power^©^ statistical software (version *3.1.9.2*). The input assumptions for the sample size calculation were an A priori F test with a 5% margin of error at 95% confidence level. Following this, the minimum sample size required to sufficiently power the study (at 80%) assuming a 100% response rate and no dropout is approximately 70 per community, making a total of 210 participants. However, assuming a 20% non-response rate, the number of participants per community is 84. This will make a total effective sample size of about 250 participants for the study.

##### Administration of Survey Questionnaire

Using a standard questionnaire, sociodemographic data including sex (male or female), age (18–25, 26–35, 36–45, 46–55, and ≥ 56 years), ethnicity, level of education, occupation, and household composition of respondents were collected. Respondents were asked about their previous history of vaccination against other flu-like diseases such as influenza, and whether they intend to accept the COVID-19 vaccination. Respondents who accepted or declined the acceptance of COVID-19 vaccine were asked further questions to determine the push and pull factors toward COVID-19 vaccination. Data collection was done through face to face interview (in-person and virtual video platforms) and digitally using an online survey tool (Google forms).

##### Determination of Vaccine Hesitancy

The most widely used model in explaining and developing strategies to overcome vaccine hesitancy is the 3-Cs model developed by the SAGE Working Group on Vaccine Hesitancy from the World Health Organization (13). The three concerns that influence vaccine acceptance, hesitancy, delayal or refusal are as follows:

- Confidence (trust in the effectiveness and safety of vaccines).
- Complacency (perceived low risk without vaccination/view that vaccines were unnecessary).
- Convenience (accessibility/availability and lack of understanding).

As part of the survey questionnaire, this study sought to measure the confidence (level of trust in the safety and effectiveness) of respondents from these communities in the COVID-19 vaccines or vaccination programme in Amsterdam, The Netherlands. The level of trust was answered using a 10-choice scale ranging from a very low level (one) to a very high level (ten). The study also sought to measure the level of complacency by asking respondents if they thought it was important for everyone to take vaccine and whether they perceived COVID-19 as a risk to their health or wellbeing. In terms of convenience, respondents were asked if they were aware about the availability of COVID-19 vaccines in Amsterdam and whether they were planning to take the vaccine.

#### Qualitative (Ethnography) Procedures

##### In-depth Interviews

Respondents who agreed were invited for additional in-depth interviews to determine the sociocultural factors driving their decison-making toward the COVID-19 vaccine acceptance or hesitancy. In-depth interviews (IDI) were conducted simultaneously alongside the collection of the survey data with the respondents. During the IDIs, the questions that generated further elaborations from the respondents were probed to investigate reasons for any discrepancies between data from the survey and what people do or say. In addition, this study explored further to understand the choices people made and the motivations behind those choices. This was a mechanism to ensure accurate data on the sociocultural beliefs and provide an understanding of the factors behind the choices people made. The IDIs were also used to discuss *immediate past* practices of the survey respondents that informed their current behavior, decisions, knowledge and opinions. This allowed for the researchers to link the choices of the respondents.

##### Participant Observation

Participant observation was a continuous element throughout the data collection procedure. During data collection, a considerable time was spent in places that Amsterdam residents of Ghanaian or Surinamese background pointed out as the most popular public places they visited for diverse reasons. The average number of people going there each day was also observed. The research team also visited some churches on different Sundays (most common day of worship), to observe the interaction between congregants present. Besides generating important contextual information, participant observation enabled the building of rapport with respondents but also generated conversations on prevention methods that were difficult and/or easy to adhere to. Most of the data were completed through face-to-face or use of video call interviews and that enabled the respondents to seek further clarification on some questions they did not completely understand. At all times, both the respondent and interviewer had their face/nose mask on and maintained a physical distance of 1.5 m from each other.

#### Collection of Ethnographic Data and Its Validation

Through a systematic inquiry, attempts were made by the researchers to consider all matters sensitive to the Ghanaian-Dutch and Surinamese-Dutch communities regarding the objective of this survey study. To assure the quality of the collected data, the questions were prepared first in English and then translated into the Dutch or Twi languages by the researcher for respondents who could not adequately understand or express themselves in English. Appropriate modifications such as wording, changing terms, rephrasing for better understanding, deleting, and adding some information for clarity were made on the tool accordingly. The researchers closely observed and monitored data collection.

### Data Analysis

The data collected through paper questionnaires and Google Forms were entered into excel and exported for analysis using SPSS software (SPSS Inc.). The descriptive proportions of participants who used each common source to obtain information about COVID-19 were presented in terms of number and percentage. The survey aimed to collect 50 responses from the Ghanaian-Dutch and each of the Surinamese-Dutch (Afro and Hindustani) communities. Putting together a representative sample of the Ghanaian-Dutch and Surinamese-Dutch residents in Amsterdam was not possible within the limited time and budget for this study. However, as previous research works have shown and this present study shows, efforts directed at specific groups and focus on their unique concerns were more effective than broad messages directed to the whole population (23, 24). Due to the method of sampling, some results and analysis are generalizable to the various age, occupational and ethnic groups who make up the sample, but this is not a representative cross-sample of the three communities in Amsterdam.

## Results

### Demographic Characteristics of Study Respondents

The demographic characteristics of the study respondents are presented in Table 1. At the end of the survey, a total of 160 responses for the survey and 36 IDI were collected through face-to-face, telephone and online interviews. There were more male respondents 86 (53.8%) compared to females 74 (46.2%). In both the Afro and Hindustani Surinamese-Dutch groups, there were more male respondents than the female respondents. However, among the respondents from the Ghanaian-Dutch community there were more females 30 (52.6%) compared to the males. Respondents belonging to the 18–25 years constituted the lowest proportion 25 (15.6%), while those in the 36–45 years had the highest proportion 37 (23.1%), with a median age range of all the respondents between 36 and 45 years.

**Table 1:** Sociodemographic characteristics of respondents from the Ghanaian-Dutch, Afro and Hindustani Surinamese-Dutch communities in Amsterdam.

**Variable**	**Ghanaian-Dutch**	**Afro Surinamese-Dutch**	**Hindustani-Dutch**	**Percent** **(%)**
**Number of respondents**	57	54	49	
**Sex**				
Male	27 (47.4%)	28 (51.9%)	31(63.3%)	53.8
Female	30 (52.6%)	26 (48.1%)	18 (36.7%)	46.2
**Age**				
18–25	8	10	7	15.6
26–35	8	9	13	18.8
36–45	15	12	10	23.1
46–55	14	10	9	20.6
≥56	12	13	10	21.9
**Level of education**				
Doctoral	2	1	3	3.8
Masters	10	8	6	15.0
Bachelor	13	18	13	27.5
Senior/Vocational/technical	26	23	20	43.1
Junior high school	1	2	4	4.4
Primary or elementary	5	1	3	5.6
None	0	1	0	0.6
**Area of occupation**				
Healthcare	8	6	7	13.1
Education	4	5	9	11.3
Transport/Construction	4	4	4	7.5
Hospitality/catering	10	5	8	14.4
Administrative /IT	3	9	5	10.6
Religious (pastoral, etc.)	2	1	3	3.8
Student	5	6	2	8.1
Unemployed/Retired	8	6	7	13.1
Others/Prefer not to answer	13	12	4	18.1

All but one of the respondents had some level of formal education ranging from primary school to doctoral degrees. The single most popular employment sector for majority of the respondents was in the hospitality 23(14.4%), followed closely by healthcare 21(11.3%), and the unemployed or retired category 21(13.1%). A total of 29 (18.1%) respondents were either employed in other non-formal sectors or preferred not to answer. Table 2 shows that majority of respondents' household were composed of 5 or more people 43(26.9%), followed by those that had 2 persons 42 (26.3%) and 3 persons 27 (16.9%). There were 61 individual respondent's homes with 2 adults living together and that constituted the highest proportion (38.1%).

**Table 2:** Household characteristics of study respondents.

**Variable**	**Ghanaian-Dutch**	**Afro Surinamese-Dutch**	**Hindustani-Dutch**	**Percent** **(%)**
**Total Household composition**				
1	5	8	13	16.3
2	9	14	19	26.3
3	12	10	5	16.9
4	5	9	6	12.5
≥ 5	24	13	6	26.9
Prefer not to answer	2	0	0	1.3
**Adults (≥18years) in household**				
1	7	12	14	20.6
2	12	25	24	38.1
3	19	7	4	18.8
4	11	6	6	14.4
≥ 5	6	2	1	5.6
Prefer not to answer	2	2	0	2.5

### Compliance Rate of Study Respondents to Previous Vaccinations Against Other Flu-Like Diseases (Influenza)

All respondents were asked if they had previously been vaccinated against other flu-like diseases such as Influenza. In the Ghanaian-Dutch community, 45 out of the 55 (81.8%) respondents answered “Yes”. Out of the 54 respondents to this survey from the Afro Surinamese-Dutch community, 44 (81.5%) had accepted previous vaccines to other flu-like diseases. Among the Hindustani-Dutch respondents 40 (81.6%) out of 49 had also previously been vaccinated against other Flu-like diseases (Table 3).

**Table 3:** Response on compliance to previous vaccinations against other flu-like diseases.

**Variable**	**GD**	**ASD**	**HD**
N	55	54	49
**Previous vaccines taken against other flu-like disease**			
Yes	45	44	40
No	10	10	9
**Driving factors toward accepting vaccines against other flu-like disease**			
Mandatory government policy	18	19	15
Proven safety and effectiveness	25	20	22
Religious or ideological belief	0	0	0
Lack of effective medication or treatment	3	3	6
Expensive treatment without vaccination	3	2	6
Protection from a deadly disease or ill-health	11	11	7
Other	2	3	3

#### Factors That Promoted Compliance to Previous Vaccination Programmes Against Other Flu-Like Diseases

Among the 44 Afro Surinamese-Dutch respondents who took previous vaccine against other flu-like diseases, 20 (45.5%) reported that they took the vaccine because they considered it was safe and effective (Table 3). Meanwhile, 19 (43.2%) took the vaccine because it was mandatory (government policy) and part of a nationwide vaccination programme. However, beyond mandatory and perceived safety or efficacy, some respondents 11 (25%) also felt convinced that the vaccine provided protection against a deadly disease. Among the Ghanaian-Dutch respondents the main driving factors for accepting previous vaccine against other flu-like diseases were based on the proven safety and effectiveness of the vaccines (55.6%) and because it was mandatory or government policy (40%). Similarly, for the Hindustani-Dutch community, most of the respondents also accepted previous vaccines against other flu-like diseases because of the proven safety and effectiveness of the vaccines (55%) and because it was mandatory or government policy (37.5%) as shown in Table 3. No respondent reported from the three ethnic groups indicated that religious or ideological belief influenced them toward or against taking the other flu-like vaccines (Table 3).

### Determining Factors for Uptake of COVID-19 Vaccine

All the respondents who took part in this study were asked if they had already taken the COVID-19 vaccine. Only 2 representing 3.6% out of the 55 respondents from the Ghanaian-Dutch (GD) community had taken at least one dose of the vaccine. Among the Afro Surinamese-Dutch respondents (ASD), only 7 (13%) had also taken at least one dose of the vaccine while a further 7 (14%) out of the 49 Hindustani-Dutch (HD) respondents had taken the vaccine (Table 4). Furthermore, respondents in this study were asked if they were planning to take the COVID-19 vaccine. The results showed that the number and percentage of respondents who were likely to take the vaccine among the Ghanaian-Dutch, Afro Surinamese-Dutch and Hindustani-Dutch were 26 (47.3%), 26 (48.1%) and 23 (47%), respectively.

**Table 4:** Perception and acceptance of COVID-19 vaccination.

**Variable**	**GD**	**ASD**	**HD**
**Have you already taken a COVID-19 vaccine shot?**			
*Yes*	2	7	7
*No*	53	47	42
**Are you planning to take the COVID-19 vaccine shot?**			
*Yes*	26	26	23
*No*	16	11	13
*Undecided*	13	17	13
**What do you think about the COVID-19 vaccine?**			
*It is effective and safe*	10	7	13
*I don't trust the effectiveness and safety*	25	18	25
*Conspiracy by the government to control us*	1	2	5
*It is important for everyone to take it*	16	26	22
*It is not important*	1	0	4
*Other/prefer not to answer*	5	6	4

There were 16 (29.1%), 11 (20.4%) and 13 (26.5%) respondents from the Ghanaian-Dutch, Afro Surinamese-Dutch and Hindustani-Dutch, respectively, that indicated they would not take the COVID-19 vaccines. A further 13 (23.6%), 17 (31.5%) and 13 (26.5%) of respondents from the Ghanaian-Dutch, Afro Surinamese-Dutch and Hindustani-Dutch were undecided about taking the vaccine (Table 4). In summary, the willingness to take the COVID-19 vaccine was relatively similar among respondents from the Ghanaian-Dutch and Afro Surinamese-Dutch respondents. However, the highest proportion of respondents who are undecided about taking the vaccines are from the Afro Surinamese-Dutch community. The data collection for the Ghanaian-Dutch respondents were completed on 14th February, 2021 and at this time the total number of people who had received at least one dose of COVID-19 vaccine in the Netherlands was 694,075 representing 4.0% of the population.

Generally, the willingness to take the COVID-19 vaccine appeared to be dependent on age groups and gender. The dependence on occupational status was not clearly determined in this study. The level of education was evenly distributed among the groups and showed no effect.

**Figure 1:**
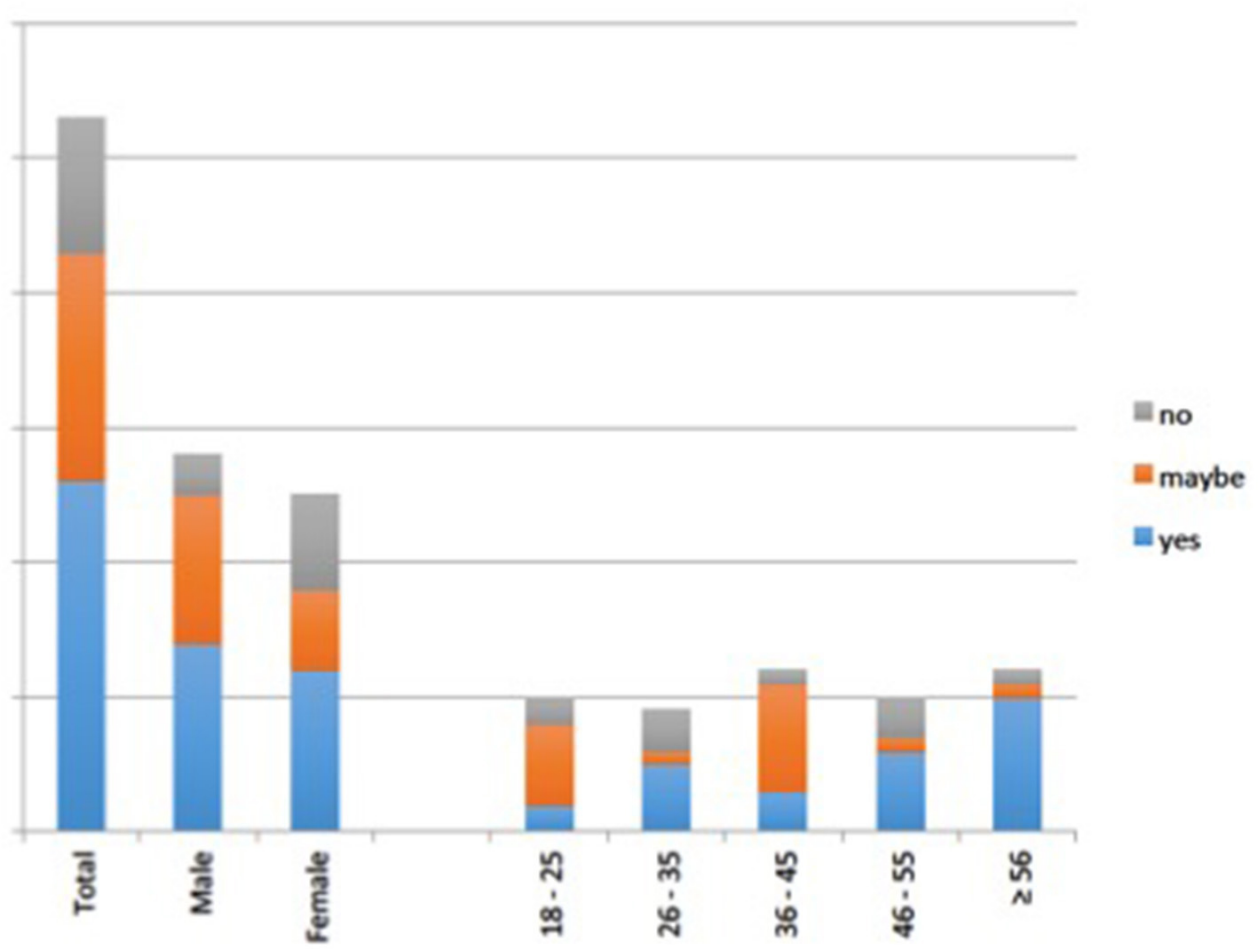
Willingness by respondents to take COVID-19 vaccination against gender and age.

In specific terms with regards to age, although older respondents (46 years or more) were more likely to say they would be vaccinated, younger respondents, age 26–35 were also more likely than those younger than them (18–25 years) or immediately older than them in the 36–45 age group (Figure 1). In terms of gender, males were significantly more likely to agree to be vaccinated than the females. The relationship between Level of Education and willingness to be vaccinated was not linear. However, respondents with a high/vocational school education formed a large proportion of those who were more willing to be vaccinated. In terms of occupation, those who showed willingness to be vaccinated include students, unemployed, self-employed and retired personnel. For most students, the willingness to get vaccinated was largely dependent on their eagerness to focus on their education with limited interruptions while the self-employed people who traveled for their businesses favored getting vaccinated. Interestingly, healthcare workers were the least willing to be vaccinated among the various occupations.

### Perceptions and Assessment of Vaccine Hesitancy

The result on perception and assessment of vaccine hesitancy is presented under complacency, confidence and convenience.

#### Complacency (Perceived Risk and Significance of Vaccination)

All respondents were asked the question “do you perceive COVID-19 crisis as a threat to your personal health or well-being?” Out of a total of 55 respondents from the Ghanaian-Dutch, 40 representing 72.7% perceived COVID-19 as a threat to their personal health or wellbeing. Out of 52 respondents from the Afro Surinamese-Dutch community, 37 representing 71.2% answered “yes” indicating that COVID-19 had negatively affected their wellbeing. From the Hindustani-Dutch community, 42 out of 49 respondents (86%) also perceived COVID-19 as a threat to their personal health or wellbeing. Many of the people affected by the pandemic explained their choice was based on the distressing effects of the COVID-19 on people in their families and communities' health or even loss of family, friends and neighbors. Others talked about it mainly because of the financial hardships that accompanied because they lost their jobs or were unable to pay for bills. For some, their businesses did not run fully and led to accumulated debts. All of these had resulted in a psychological toll on their lives of many respondents. Talking to 53-year-old Anish (male), he explained that the Hindustani community in Amsterdam is perhaps the worst hit with COVID-19 fatalities, saying….

Due to the lockdown, many of us were not seeing each other. We socialize less but after that I heard people telling me other people I knew had died from corona. You ask of people you used to play cards with and then you are told they died, so very sad. I even read an article that said that a lot of Hindoestaanse have died from COVID. It is very serious. And most of us apart from age have some other sicknesses; I have diabetes. When age and these heart conditions come together plus corona then we are heading toward a lot of death

Among the Ghanaian-Dutch respondents, 27% (16) indicated that it is important for everyone to take the vaccine. Among the Afro Surinamese-Dutch respondents, majority of them 26 (48.1%) responded that “I think it is important for everyone to take it. A high proportion of respondents from the Hindustani-Dutch community, 22 (44.9%) think that it is important for everyone to take the vaccine”. Compared to the other two groups, the Hindustani-Dutch community had a slightly higher proportion of respondents who think the vaccine is not important 4 (8.2%). None of the respondents from the Ghanaian-Dutch or the Afro Surinamese-Dutch indicated that “It is not important”.

#### Confidence (Trust in the Effectiveness and Safety of Vaccines)

To gauge the perception toward the current COVID-19 vaccines, respondents were asked “what do you think about the COVID-19 vaccine?” The responses received across the different ethnic communities are presented below in Table 4. The majority of respondents 25 (43%) from the Ghanaian-Dutch community reported that they do not trust the effectiveness and safety of the COVID-19 vaccine and only 10 (17%) of the respondents believed that the vaccines are effective and safe. Only 1 respondent indicated it was a conspiracy by the government to control the people. Among the Afro Surinamese-Dutch respondents, 18 (33.3%) also answered that “I don't trust the effectiveness and safety” while 7 (13%) indicated that “I think it is effective and safe”. There were 2 (3.7%) respondents who considered the introduction of the COVID-19 vaccines was a “conspiracy by the government to control us”. The largest proportion of respondents of the Hindustani-Dutch community, 25 (51%) indicated that they “do not trust the effective and safety of the vaccine”. A further 13 (26.5%) think that the “vaccines are effective and safe”. A few of the respondents 5 (10.2%) from the Hindustani-Dutch community also considered the vaccine as a government policy to control the citizens.

In general, the proportion of respondents who indicated that they do not trust the effectiveness and safety of the vaccine were highest among the Hindustani-Dutch, followed by the Ghanaian-Dutch and lastly the Afro Surinamese-Dutch communities.

##### Scaling of Confidence Level in the COVID-19 Vaccines

**Figure 2:**
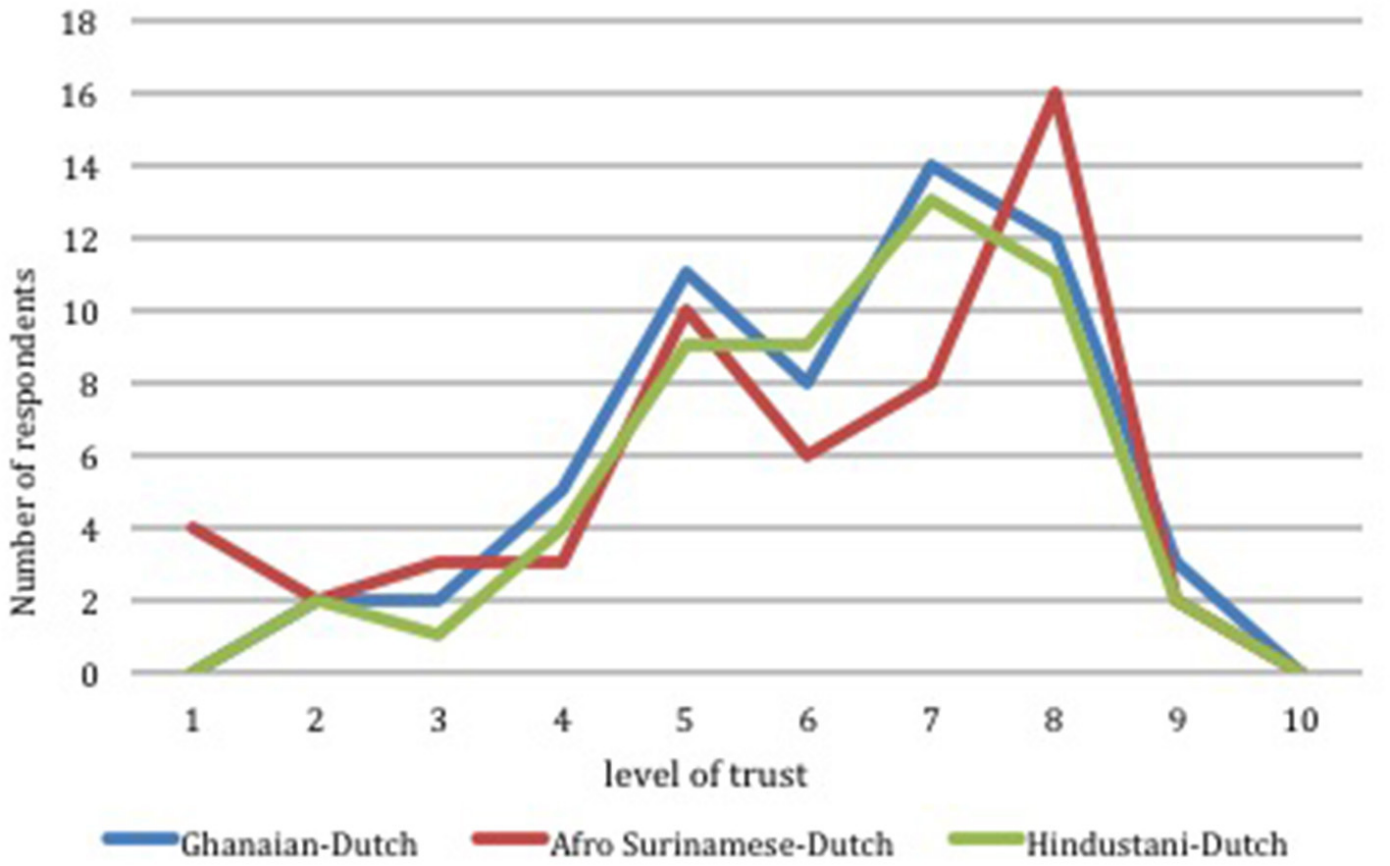
Level of trust in the safety and effectiveness of COVID vaccines across 3 different ethnic groups in Amsterdam.

The results presented in Table 4 above shows that trust in the safety and effectiveness of the COVID-19 vaccines was a major concern for most of the respondents in this survey. This survey therefore attempted to examine the level of confidence (trust in the safety and effectiveness) of the vaccines by asking the question “how would you rate your level of trust in the COVID-19 vaccine to offer protection against severe disease?” A 10-grade scale was used to assess the level of trust by choosing from very low (one) to very high (ten). Figure 2 shows the illustration of the level of trust as reported by all the 160 respondents from the various communities. The peak level of trust in the COVID-19 vaccines as expressed by respondents for the Ghanaian community was 7 out of 10 (range 2–9). Among the Afro Surinamese-Dutch community, 16 (29.6%) out of the 54 respondents scored their level of trust in the COVID-19 vaccines as 8/10 (range 1–9). For respondents from the Hindustani-Dutch, the peak level of trust was 7 (range 2–9). Thus, there was a general skewness above the midpoint level of confidence, an indication of a high level of trust in the effectiveness of the vaccines to protect them.

#### Convenience (Accessibility/Availability and Lack of Understanding)

This survey considered the level of awareness and concerns of the respondents of the on-going COVID-19 vaccination. A total of 53 (96.4%) out of the 55 respondents from the Ghanaian-Dutch community were aware of the availability or that COVID-19 vaccination has started in Amsterdam and across the Netherlands. Among the Afro Surinamese-Dutch respondents, nearly all (96%) of the 52 respondents indicated they were aware about the availability or that COVID-19 vaccination had started in Amsterdam. Out of a total of 49 responses received from the Hindustani-Dutch community, 45 representing 92% respondents indicated their awareness about the COVID-19 vaccination programmes being implemented across the metropolitan region in Amsterdam and across the Netherlands. Those who said they did not know were of the view that it was the trial period and perhaps the large-scale vaccination would start later.

A total of 35 out of the 55 (64%) respondents from the Ghanaian-Dutch community answered “yes” to the question “do you have any concerns about taking the COVID-19 vaccine?”. In addition, 32 (62%) of the 52 respondents from the Afro Surinamese-Dutch community to the question “do you have any concerns about taking the COVID-19 vaccine” answered “yes”. Among the Hindustani-Dutch, 32 out of 48 respondents (67%) indicated that they also had concerns about the COVID-19 vaccines. A significant proportion of the respondents indicated they fear that the COVID-19 vaccine may either be harmful or have severe side effects. Some respondents were simply concerned that the vaccine was discovered too quickly. These were followed by concerns such as the vaccine is Government policy to use/introduce vaccine passport to control us, lack of trust in the science behind the vaccine development and lack of trust in the source/manufacturer of the COVID-19 vaccine.

Taking urgency and cautiousness together, participants' views and interest in the COVID-19 vaccination were mixed. A close observation revealed that people were carefully engaging with the experiences and stories of others (raising tensions but also quest for more information) regarding the COVID-19 vaccination. As noted by Sahoed, a 57 years old Afro Surinamese-Dutch man;

It would be an understatement to say I rushed to my huisart to get my prick two days ago. Rolling down my sleeve after my prick, I felt as if I had been fully protected and can start hugging and visiting friends again. But my joy was not for long when I realized many of my friends and family did not want to get the prick or when they wanted they could hardly get it because it was not their time yet. The decision is a hard one to think of. But I am happy I am out of the dangerous zones.

### Factors Likely to Influence Acceptance of the COVID-19 Vaccine

Majority of the respondents 37 (56%) from the Ghanaian-Dutch community indicated their preparedness to take the COVID-19 vaccine when it is proven to be safe and effective. Other factors that could influence the acceptance of the COVID-19 vaccine are personal understandings 12 (18%), travel requirement 7 (11%), and trust in government policy 5 (8%). Only a small proportion of respondents indicated their willingness to accept the COVID-19 vaccine when it is recommended by their family and friends 2 (3%), or by their employers 2 (3%). The study showed that 29 (58%) among the Afro Surinamese-Dutch respondents indicated that the most influential factors that can influence their willingness to take the vaccine is when there is a mandatory introduction of a vaccine passport by the government. The availability of sufficient scientific data to prove a high vaccine efficacy (proven safety and effectiveness) and mandatory vaccination as a travel requirement account for 40 and 36%, respectively. Recommendations by family and friends 8 (16%), or by respondents' general practitioners 3 (6%) or by employers 2 (4%) were other factors that could influence acceptance of COVID-19 vaccination. Among the Hindustani-Dutch community, the scientific proven high vaccine effectiveness and safety could encourage about 41.3% of the respondents to accept the vaccine. In addition, this study shows that 32 (69.6%) of the respondents appear likely to accept the vaccine when the introduction of vaccine passports is made mandatory by the government. Other major factors that could drive the likelihood of accepting the COVID-19 vaccine include travel requirement for vaccination (28.3%), the probability of minor side effects (26.1%) and recommendation by family and friends (15.2%).

## Discussion

### Perception of the COVID-19 Vaccination

National polls conducted in the Netherlands before the roll out of COVID-19 vaccination programmes began suggested that about 40% of the population were hesitant to receive COVID-19 vaccination (25). However, the public perception and response to the COVID-19 vaccination among some minority ethnic groups remain undetermined.

This present study revealed that some respondents think that the COVID-19 vaccine is important because of the significant effect of previous vaccinations toward other contagious diseases while others had concerns about how the COVID-19 vaccines will come to shape almost every area of their lives either granting them access or not to certain spaces and settings.

The findings from this present study show that public or individual perceptions about the COVID-19 are not homogenous but appear to vary widely among people from the minority ethnic communities in Amsterdam. More so it shows that there are wide sociocultural and demographic status variations in the perception or opinions and understandings among individuals within the same community or among the three selected minority ethnic groups, showing the differences concerning their vulnerability or reluctance to engage with COVID-19 vaccination programmes (26).

Even before the pandemic, public health agencies around the world were struggling to counter increasingly sophisticated efforts to turn people against vaccines in general (27). Coronavirus vaccines seem to face additional hurdles, especially the lack of a long-term safety record as well as inadequate stories of others that had received the vaccine especially at the time of data collection (28). The frenetic pace of vaccine development has played into the concerns raised by respondents in this study as seen in other research works (29). Even those who are eager to receive their shots have been worried that the vaccine could be ineffective or have harmful side effects.

Lupton's [(30), p. 7] underscores that risk perception “is intersubjective produced through social relations,” and so through observation and interviews we can better understand the “ways of making sense of situations, naming responses, part of the diverse cultural meaning systems that we use to try to understand the world.” One significant observation in this present study was the claims that “one [could] boost their immune system or cure COVID-19” by taking vegetables and fruits including vitamin C and hot herbal teas. When people have perceptions that taking certain medications, herbs or “immune boosters” can protect them against the virus or even heal them, it could have huge impact on their decision-making regarding taking vaccines. The implication is also that such people may hesitate, refuse or delay in taking up the COVID-19 vaccine.

Besides the safety concerns, others were concerned that the different vaccines may be a conspiracy by the governments all over the world to control population. The notion that the disease is a conspiracy involving the governments and philanthropists has an effect on the reception of the COVID-19 vaccine. Many of our study respondents have relied on social media posts to create the psychological habits that makes them think doing nothing is safer than taking action. The Health Belief Model (HBM) and its conceptual framework have been very instrumental for evaluating some of these psychological habits, beliefs and attitude toward some major vaccines including those against influenza, swine flu and COVID-19 (31–33). However, our present study did not examine the COVID-19 acceptance or hesitancy factors based on the constructs of the HBM. This is because as our findings show using such models alone to evaluate health decisions by people could be inadequate as their decisions are influenced also by their current living situation, advice from friends or experts, past experiences and embodied routines (34).

More so, in a media environment that favors speed, emotion, and memorable stories that can easily circulate, it is essential that the accurate and reliable information on vaccine safety and effectiveness is clearly available. Thus, it may be important for the media as well as the Dutch public health agencies to focus on publishing more diverse stories with different outcomes and avoid “the danger of single story” that portrays doom. The stories people see, hear or read have an impact on how they decide on important issues that concerns their live and that of others. The Dutch public health service could follow some of the current initiatives have pioneered a more story-based approach such as the National Human papillomavirus (HPV) Vaccination Roundtable, which promotes vaccination against the human papillomavirus, a leading cause of cervical cancer, through YouTube videos of women who survived cervical cancer to share their story with other women (35).

### Determinants of COVID-19 Vaccine Among Minority Ethnic Groups in the Netherlands

Emerging evidence from some of the countries with most successful early roll out of COVID-19 vaccination for its population including Israel, Chile, United States of America and the United Kingdom shows that the uptake of vaccines among minority groups is low (10). Findings from this present study shows that <4, 13 and 14% of respondents from the Ghanaian-Dutch, Afro Surinamese-Dutch and the Hindustani Surinamese-Dutch communities, respectively, had taken at least one dose of the COVID-19 vaccines. As of 7th February 2021 when data collection for the Ghanaian-Dutch community was concluded, only 694, 075 (4%) of the Dutch population had been vaccinated. Thus, it is difficult to assume that there was a higher vaccine hesitancy rate among the Ghanaian-Dutch community as the proportion seem to correspond with that of the general population at the time. However, as at 25th April 2021 when data collection for the Afro Surinamese-Dutch and Hindustani-Dutch ended, a total of 3,930,671 people in the Netherlands had received at least one dose of the vaccine representing 22.7% of the Country's population (36). This suggests that the uptake of the COVID-19 vaccines may be low among these two minority ethnic groups compared to the national average. This finding shows the need to pay particular attention to where there are divergences among population groups.

In a similar study in the UK, it was found that there was “substantial divergence in the uptake of vaccine” as the proportion of those vaccinated was lowest at 20.5% among the black population of Bangladeshi and Pakistani descent (37). The findings from this present study and others raises great public health concern since people from these minority ethnic groups seem to have the highest COVID-19 hospitalization, and mortality rates in the Netherlands (38, 39) including higher rates of job loss among others. In a recent study, the Ghanaian-Dutch population had a high seropositive prevalence for COVID-19 and was found to be associated with age and large household composition (40). Our findings also showed a high proportion of respondents across the three ethnic groups live in homes with high household sizes and that could pose a great public health threat if efforts to improve vaccination rate are not implemented to protect individuals and their households.

The findings from this study revealed that the percentage of respondents who were likely to take the vaccine among the Ghanaian-Dutch, Afro Surinamese-Dutch and Hindustani-Dutch were 47.3, 48.1 and 47%, respectively. Our findings show that the male respondents were more likely to accept the vaccine than the female respondents. Age-dependent analysis also indicated that the likelihood to accept the COVID-19 vaccination was linearly correlated with increasing age with the highest acceptance among respondents aged 56 years or older. This finding is similar to other findings from Israel, which showed the highest uptake of COVID-19 vaccine among persons aged 50 years or older (11).

Consequently, there were nearly 23.6, 31.5 and 26.5% of respondents from the Ghanaian-Dutch, Afro Surinamese-Dutch and Hindustani-Dutch, respectively, who were undecided about taking the vaccine. A further 29.1, 20.4 and 26.5% of respondents from the Ghanaian-Dutch, Afro Surinamese-Dutch and Hindustani-Dutch, respectively, opted not to take the COVID-19 vaccines. As suggested by earlier studies the main factors for the low uptake of COVID-19 vaccines include perceived lower risk of infection, socially disadvantaged groups (41), socioeconomic status, level of education, inconvenience and access barriers (42). In addition, this present study show that other factors for the low uptake of COVID-19 vaccines is the lack or low level of confidence in the safety or efficacy of the vaccines across all ethnic, demographic and occupational groups (43, 44). In this present study, this confidence is grounded not in the reputation of any single manufacturer of vaccines, but on the scientific method that is required by manufacturers to demonstrate that a vaccine works and is safe.

Our findings revealed that respondents showed trust and confidence in the ability of the COVID-19 vaccine to protect them but were skeptical about the notion of “good” and “bad” COVID-19 vaccines. This is in reference to the news in the media about certain types of vaccines causing some rare form of thrombosis in persons who took that vaccination (45–47). As evident by this, the fear of the COVID-19 vaccine causing harmful or severe side effects (90.6%) appears to be the major concern respondents had about the COVID-19 vaccines. The finding from this study supports the findings by the UK Scientific Advisory Group for Emergencies (SAGE) ethnicity sub-group review in December 2020 that looked at the factors influencing COVID-19 vaccine uptake among minority ethnic groups (42, 48).

The causes of vaccine hesitancy often correspond strongly with past research around public confidence in various vaccines (12, 49, 50). This study has demonstrated that COVID-19 vaccine decision-making of some ethnic minorities in the Netherlands may hinge on issues, concerns and anxieties. A vitriolic public (health) message or representations that single out minority ethnic groups may only run the risk of damaging their relations with public health and other health institutions or workers, and can be avoided by better understanding the processes of vaccine decision-making.

The main limitation of this study was that data collection was conducted at the period when the COVID-19 infection rate in the Netherlands had reached its second peak (second wave). The imposition of strict lockdown and other mitigation measures such as “work from home” and “ban on mass meeting” made it difficult to recruit a high number of study participants. As a result, participant observations were limited to participants who were willing to invite us into their homes, meetings in addition to open public spaces.

## Conclusion

The COVID-19 pandemic has created the need of developing a prioritized set of vaccine recommendations and communications as well as varying them among different communities. This survey study provided a systematic evidence of factors associated with participants' decision making regarding COVID-19 vaccination among the Ghanaian-Dutch, Afro Surinamese-Dutch and the Hindustani-Dutch communities in Amsterdam. The findings show that vaccine choice and likelihood of self-reported willingness to receive a vaccine were associated with vaccine efficacies. As shown in this study, the scientific demonstration of high vaccine efficacy could encourage about 41.3% of the respondents to accept the vaccine. In addition, 69.6% of the respondents appear likely to accept the vaccine when the introduction of vaccine passports is made mandatory by the government. Other major factors that could drive the likelihood of accepting the COVID-19 vaccine include travel requirement for vaccination (28.3%), the probability of minor side effects (26.1%) and recommendation by family and friends (15.2%). The analyses in this study provide insights about the groups or factors that are likely to be associated with vaccine hesitancy, so as to inform public health efforts to communicate effectively about the COVID-19 vaccine.

## Data Availability Statement

The raw data supporting the conclusions of this article will be made available by the authors, without undue reservation.

## Ethics Statement

The studies involving human participants were reviewed and approved by AISSR Ethics Board. Written informed consent for participation was not required for this study in accordance with the national legislation and the institutional requirements.

## Author Contributions

DA-B and AZB conceived and planned the experiment, carried out the data collection, and supervised the findings of this work. DA-B, AZB, KBO, and AK verified the analytical methods and discussed the results and contributed to the final manuscript. All authors contributed to the article and approved the submitted version.

## Conflict of Interest

The authors declare that the research was conducted in the absence of any commercial or financial relationships that could be construed as a potential conflict of interest.

## Publisher's Note

All claims expressed in this article are solely those of the authors and do not necessarily represent those of their affiliated organizations, or those of the publisher, the editors and the reviewers. Any product that may be evaluated in this article, or claim that may be made by its manufacturer, is not guaranteed or endorsed by the publisher.
